# Understanding the Tradition and Impact of VA Innovation to Geriatric Care

**DOI:** 10.1093/geroni/igaf122.481

**Published:** 2025-12-31

**Authors:** Lea Kiefer, Caroline Madrigal, Caitlin Kappler, Katherine Hall, Katherine Ritchey, Nihar Shetty, Anne Wang, Priya Bapna

**Affiliations:** Michael E. DeBakey VA Medical Center, Houston, Texas, United States; VA Boston Healthcare System, Boston, Massachusetts, United States; Durham Veterans Affairs, Durham, North Carolina, United States; Veterans Health Administration, Durham, North Carolina, United States; Department of Veterans Affairs, Seattle, Washington, United States; Michael E. DeBakey VA Medical Center, Houston, Texas, United States; Michael E. DeBakey VA Medical Center, Houston, Texas, United States; Michael E. DeBakey VA Medical Center, Houston, Texas, United States

## Abstract

The Department of Veterans Affairs (VA) has been a leader in geriatric care since the aftermath of World War I, pioneering specialized programs to meet the needs of aging Veterans. In 1975, Congress established the Geriatric Research, Education, and Clinical Centers (GRECCs). There are currently 20 GRECCs located in VA medical centers across the country, and each is connected with an academic medical institution. The GRECCs are the engines of discovery and innovation, and have been testing and developing models of care nearly a decade before similar programs/initiatives emerged in Medicare. This commitment deepened in 1990 with the creation of the Geriatrics and Extended Care (GEC) service line, which expanded access to comprehensive care focused on preventing disability and promoting independence for older adults through evidence-based, patient-centric programs and services for Veterans and their families/caregivers. VA’s sustained investments in geriatric care resulted in numerous clinical innovations that became standard practice, not only within the VA system but also in broader healthcare settings. Many programs predated similar private sector initiatives, exemplifying VA’s proactive approach to meeting the evolving needs of the nation’s aging population. VA has a rich history of pioneering advancements in geriatric care and has played a crucial role in translating research into evidence-based practices. By examining the evolution of VA’s geriatric initiatives and their collaboration with community-based initiatives, we can see how VA continues to shape the future of geriatric care –leveraging research, education, and clinical innovation to meet the evolving needs of the Veteran population.

